# 2,2′-(Diselane-1,2-di­yl)dinicotinamide *N*,*N*′-dimethyl­formamide disolvate

**DOI:** 10.1107/S1600536810015047

**Published:** 2010-04-30

**Authors:** Aixia Feng, Ying Xu, Xuehong Wei

**Affiliations:** a School of Chemistry and Chemical Engineering, Shanxi University, Shanxi Province, People’s Republic of China

## Abstract

The asymmetric unit of the title compound, C_12_H_10_N_4_O_2_Se_2_·2C_3_H_7_NO, contains two solvent mol­ecules and two half mol­ecules of the dinicotinamide, each of which sits on a center of symmetry passing through the middle of the Se—Se bond. In each mol­ecule, the two pyridyl groups and diseleno group are approximately coplanar (r.m.s. deviations from planarity for all non-H atoms = 0.011 and 0.008 Å in the two mol­ecules). Inter­molecular N—H⋯O hydrogen bonds stablilize the crystal packing.

## Related literature

For the potential applications of organoselenium compounds in organic synthesis, as precursors for semiconducting materials and in ligand chemistry and biochemistry, see: Mugesh *et al.* (2001 ▶). For related diselenide compounds, see: Bhasin & Singh (2002 ▶); Kienitz *et al.* (1996 ▶).
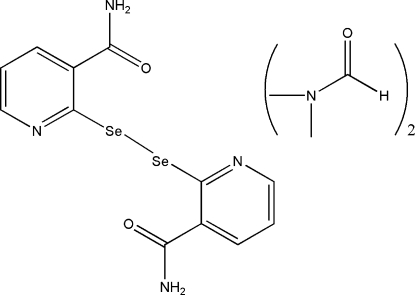


## Experimental

### 

#### Crystal data

C_12_H_10_N_4_O_2_Se_2_·2C_3_H_7_NO
*M* *_r_* = 546.34
Triclinic, 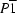

*a* = 7.6101 (17) Å
*b* = 12.318 (3) Å
*c* = 13.420 (3) Å
α = 114.175 (2)°
β = 91.017 (3)°
γ = 95.833 (3)°
*V* = 1139.3 (4) Å^3^
*Z* = 2
Mo *K*α radiation
μ = 3.28 mm^−1^
*T* = 298 K
0.30 × 0.20 × 0.20 mm

#### Data collection

Siemens SMART CCD diffractometer
Absorption correction: multi-scan (*SADABS*; Sheldrick, 1996 ▶) *T*_min_ = 0.439, *T*_max_ = 0.560
3937 measured reflections
3937 independent reflections
3359 reflections with *I* > 2σ(*I*)

#### Refinement

*R*[*F* ^2^ > 2σ(*F* ^2^)] = 0.036
*wR*(*F* ^2^) = 0.097
*S* = 1.03
3937 reflections
275 parameters
H-atom parameters constrained
Δρ_max_ = 0.38 e Å^−3^
Δρ_min_ = −0.73 e Å^−3^

### 

Data collection: *SMART* (Siemens, 1996 ▶); cell refinement: *SAINT* (Siemens, 1996 ▶); data reduction: *SAINT*; program(s) used to solve structure: *SHELXS97* (Sheldrick, 2008 ▶); program(s) used to refine structure: *SHELXL97* (Sheldrick, 2008 ▶); molecular graphics: *SHELXTL* (Sheldrick, 2008 ▶); software used to prepare material for publication: *SHELXL97*.

## Supplementary Material

Crystal structure: contains datablocks I, global. DOI: 10.1107/S1600536810015047/fl2299sup1.cif

Structure factors: contains datablocks I. DOI: 10.1107/S1600536810015047/fl2299Isup2.hkl

Additional supplementary materials:  crystallographic information; 3D view; checkCIF report

## Figures and Tables

**Table 1:** Hydrogen-bond geometry (Å, °)

*D*—H⋯*A*	*D*—H	H⋯*A*	*D*⋯*A*	*D*—H⋯*A*
N1—H1*A*⋯O4	0.86	2.09	2.946 (4)	170
N1—H1*B*⋯O3	0.86	2.03	2.869 (5)	163
N3—H3*B*⋯O4	0.86	2.10	2.919 (4)	158
N3—H3*A*⋯O1^i^	0.86	2.31	3.081 (4)	150

Symmetry code: (i) 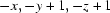.

**Fig. 1.**
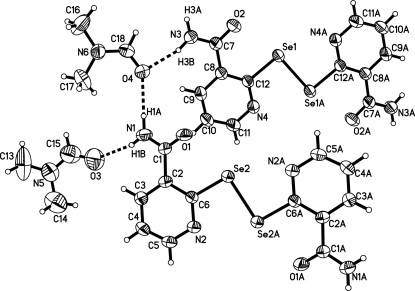
Structure showing 50 % probability.

## supplementary crystallographic information

### Comment

Organoselenium compounds have attracted much atention because of their potential applications in organic synthesis, precursors for semiconducting materials, ligand chemistry and biochemistry (Mugesh *et al.*, 2001). During the past decade, many organoselenium compounds have been synthesized and well characterized. In contrast to alkyl, aryl, and mixed alkylaryl senium compounds, the pyridyl selenium compounds are still rare.

The unit cell contains two nicotinamide molecules and four of the solvent molecules. The asymmetric unit contains two solvent molecules and two half molecues of the nicotinamide each of which sits on a center of symmetry passing through the middle of the Se—Se bond (Fig. 1). In (I),the  two independent nicotinamides (molecule A containing  Se1 and molecule B containing  Se2) have comparable conformations. In each nicotinamide, the two pyridyl groups and the diseleno group are approximately coplanar (r.m.s. deviations from planarity for all non-H atoms are 0.011 and 0.008Å for molecules A and B, respectively while the two CONH_2_ groups are rotated out of this plane by 11.0 (5)° and 18.6 (5)° for molecules A and B, respectively. Fig. 2 shows the  sheets of molecules formed by intermolecular N-H···O hydrogen-bond interactions between the nicotinamides and neighbouring solvents with distances between 2.869 (5) and 3.081 (4) Å (Table 1).

The structure of (I) is similar to that of other diselenide compounds (Kienitz, *et al.* 1996; Bhasin and Singh 2002).The two neighbouring pyridyl groups can be brought into register by rotation about the Se—Se bond. The commonly observed approximate coplanarity of the rings and the Se—Se bonds (C—C—Se—Se or N—C—Se—Se torsion angles ca. 0°) in these molecules has been explained in terms of a minimization of Se···Se lone pair repulsion.

### Experimental

To a vigorously stirred solution of selenium powder (1.19 g, 15 mmol) and absolute ethanol (30 ml), sodium borohydride (0.40 g, 10.6 mmol) was added at 0 °C. The mixture was warmed to room temperature and stirred for 2 h. 2-Chloro-nicotinamide (1.56 g, 10 mmol) was added and stirred for 7 days. O_2_ was passed through the solution slowly for 2 h after the reaction mixture was acidfied by glacial acetic. The solvents were removed in vacuo and the residue was extracted with hot dimethyl sulphoxide (DMSO) and filtered. The filtrate was poured into water( 200 ml, cooled to 0 °C). The precipitate was separated by filtration and recrystallized from DMSO-CH_3_OH(1:2) to give the product as yellow crystals, yield: 1.56 g, 78%; m.p. 124-125 °C. ^1^H-NMR ( 300 MHz, DCCl_3_) δ (ppm): 7.27 (d, 2H), 7.81 (s, 2H), 8.14 (d, 2H), 8.32 (s, 2H), 8.48 (s, 2H), 8.65 (s, 2H);^77^Se-NMR (57 MHz, DMSO-d_6_ ) δ(ppm): 524.77.

### Refinement

(type here to add refinement details)

### Crystal data

**Table:**

C_12_H_10_N_4_O_2_Se_2_·2C_3_H_7_NO	*Z* = 2
*M**_r_* = 546.34	*F*(000) = 548
Triclinic, *P*1	*D*_x_ = 1.593 Mg m^−^^3^
*a* = 7.6101 (17) Å	Mo *K*α radiation, λ = 0.71073 Å
*b* = 12.318 (3) Å	Cell parameters from 2815 reflections
*c* = 13.420 (3) Å	θ = 3.1–27.5°
α = 114.175 (2)°	µ = 3.28 mm^−^^1^
β = 91.017 (3)°	*T* = 298 K
γ = 95.833 (3)°	Block, yellow
*V* = 1139.3 (4) Å^3^	0.30 × 0.20 × 0.20 mm

### Data collection

**Table:**

Siemens SMART CCD diffractometer	3937 independent reflections
Radiation source: fine-focus sealed tube	3359 reflections with *I* > 2σ(*I*)
graphite	*R*_int_ = 0.0000
phi and ω scans	θ_max_ = 25.0°, θ_min_ = 1.7°
Absorption correction: multi-scan (*SADABS*; Sheldrick, 1996)	*h* = −9→8
*T*_min_ = 0.439, *T*_max_ = 0.560	*k* = −14→13
3937 measured reflections	*l* = 0→15

### Refinement

**Table:**

Refinement on *F*^2^	Primary atom site location: structure-invariant direct methods
Least-squares matrix: full	Secondary atom site location: difference Fourier map
*R*[*F*^2^ > 2σ(*F*^2^)] = 0.036	Hydrogen site location: inferred from neighbouring sites
*wR*(*F*^2^) = 0.097	H-atom parameters constrained
*S* = 1.03	*w* = 1/[σ^2^(*F*_o_^2^) + (0.0416*P*)^2^ + 0.7097*P*] where *P* = (*F*_o_^2^ + 2*F*_c_^2^)/3
3937 reflections	(Δ/σ)_max_ = 0.001
275 parameters	Δρ_max_ = 0.38 e Å^−^^3^
0 restraints	Δρ_min_ = −0.73 e Å^−^^3^

### Special details

**Table:**

Geometry. All esds (except the esd in the dihedral angle between two l.s. planes) are estimated using the full covariance matrix. The cell esds are taken into account individually in the estimation of esds in distances, angles and torsion angles; correlations between esds in cell parameters are only used when they are defined by crystal symmetry. An approximate (isotropic) treatment of cell esds is used for estimating esds involving l.s. planes.
Refinement. Refinement of *F*^2^ against ALL reflections. The weighted *R*-factor wR and goodness of fit *S* are based on *F*^2^, conventional *R*-factors *R* are based on *F*, with *F* set to zero for negative *F*^2^. The threshold expression of *F*^2^ > σ(*F*^2^) is used only for calculating *R*-factors(gt) etc. and is not relevant to the choice of reflections for refinement. *R*-factors based on *F*^2^ are statistically about twice as large as those based on *F*, and *R*- factors based on ALL data will be even larger.

### Fractional atomic coordinates and isotropic or equivalent isotropic displacement parameters (Å^2^)

**Table:**

	*x*	*y*	*z*	*U*_iso_*/*U*_eq_	
Se1	0.44747 (5)	0.90079 (3)	0.43528 (3)	0.04343 (13)	
Se2	−0.00660 (5)	0.90803 (3)	0.92023 (2)	0.04228 (13)	
N1	0.1532 (5)	0.5410 (3)	0.7795 (2)	0.0635 (10)	
H1A	0.1427	0.5036	0.7093	0.076*	
H1B	0.2126	0.5139	0.8176	0.076*	
N2	0.0893 (4)	0.8848 (2)	1.1107 (2)	0.0425 (7)	
N3	0.2600 (5)	0.5278 (3)	0.3920 (3)	0.0592 (9)	
H3A	0.2117	0.4794	0.3289	0.071*	
H3B	0.2580	0.5066	0.4457	0.071*	
N4	0.5588 (4)	0.9107 (3)	0.6373 (2)	0.0486 (7)	
N5	0.3752 (5)	0.2618 (4)	0.9343 (3)	0.0708 (10)	
N6	0.0494 (5)	0.2208 (3)	0.4807 (2)	0.0551 (8)	
O1	−0.0074 (4)	0.6808 (2)	0.77632 (19)	0.0673 (9)	
O2	0.3436 (5)	0.6689 (2)	0.3326 (2)	0.0745 (9)	
O3	0.3116 (6)	0.4039 (4)	0.8778 (3)	0.1119 (15)	
O4	0.1655 (4)	0.4149 (2)	0.5403 (2)	0.0636 (8)	
C1	0.0787 (5)	0.6393 (3)	0.8283 (3)	0.0453 (8)	
C2	0.0992 (4)	0.7021 (3)	0.9497 (2)	0.0385 (7)	
C3	0.1484 (5)	0.6470 (3)	1.0157 (3)	0.0444 (8)	
H3	0.1689	0.5671	0.9840	0.053*	
C4	0.1672 (5)	0.7094 (3)	1.1279 (3)	0.0501 (9)	
H4	0.1983	0.6727	1.1728	0.060*	
C5	0.1388 (5)	0.8271 (3)	1.1708 (3)	0.0481 (9)	
H5	0.1547	0.8700	1.2463	0.058*	
C6	0.0676 (4)	0.8226 (3)	1.0022 (2)	0.0359 (7)	
C7	0.3379 (5)	0.6354 (3)	0.4068 (3)	0.0485 (9)	
C8	0.4199 (5)	0.7156 (3)	0.5172 (3)	0.0415 (8)	
C9	0.4458 (6)	0.6768 (3)	0.5990 (3)	0.0555 (10)	
H9	0.4074	0.5980	0.5869	0.067*	
C10	0.5283 (6)	0.7548 (4)	0.6982 (3)	0.0649 (12)	
H10	0.5460	0.7299	0.7539	0.078*	
C11	0.5836 (6)	0.8695 (4)	0.7131 (3)	0.0597 (11)	
H11	0.6417	0.9216	0.7797	0.072*	
C12	0.4806 (4)	0.8354 (3)	0.5408 (3)	0.0382 (7)	
C13	0.4094 (8)	0.1434 (5)	0.9066 (7)	0.132 (3)	
H13A	0.3984	0.0998	0.8285	0.198*	
H13B	0.3259	0.1059	0.9390	0.198*	
H13C	0.5273	0.1436	0.9334	0.198*	
C14	0.3803 (8)	0.3421 (6)	1.0492 (5)	0.1017 (18)	
H14A	0.3586	0.4203	1.0564	0.153*	
H14B	0.4947	0.3471	1.0833	0.153*	
H14C	0.2910	0.3120	1.0842	0.153*	
C15	0.3437 (7)	0.3039 (5)	0.8611 (4)	0.0867 (15)	
H15	0.3463	0.2508	0.7882	0.104*	
C16	−0.0269 (7)	0.1122 (4)	0.3893 (4)	0.0864 (16)	
H16A	−0.0493	0.1295	0.3270	0.130*	
H16B	−0.1361	0.0820	0.4089	0.130*	
H16C	0.0541	0.0530	0.3715	0.130*	
C17	0.0853 (7)	0.2161 (4)	0.5860 (4)	0.0791 (14)	
H17A	0.1766	0.2791	0.6280	0.119*	
H17B	0.1233	0.1400	0.5741	0.119*	
H17C	−0.0205	0.2260	0.6252	0.119*	
C18	0.0939 (6)	0.3203 (4)	0.4690 (3)	0.0571 (10)	
H18	0.0690	0.3193	0.4005	0.069*	

### Atomic displacement parameters (Å^2^)

**Table:**

	*U*^11^	*U*^22^	*U*^33^	*U*^12^	*U*^13^	*U*^23^
Se1	0.0546 (2)	0.0391 (2)	0.0405 (2)	−0.00010 (15)	−0.00515 (15)	0.02201 (17)
Se2	0.0608 (2)	0.0352 (2)	0.02975 (19)	0.00734 (15)	−0.00560 (15)	0.01226 (15)
N1	0.103 (3)	0.051 (2)	0.0322 (15)	0.0285 (19)	−0.0013 (16)	0.0088 (14)
N2	0.0567 (18)	0.0399 (16)	0.0296 (14)	0.0083 (13)	−0.0010 (12)	0.0127 (12)
N3	0.084 (2)	0.0411 (18)	0.0469 (17)	−0.0106 (16)	−0.0191 (16)	0.0181 (15)
N4	0.062 (2)	0.0385 (16)	0.0464 (17)	−0.0001 (14)	−0.0092 (14)	0.0207 (14)
N5	0.062 (2)	0.079 (3)	0.081 (3)	0.0151 (19)	0.0075 (19)	0.041 (2)
N6	0.074 (2)	0.0438 (18)	0.0476 (18)	0.0091 (15)	0.0171 (16)	0.0181 (15)
O1	0.113 (2)	0.0510 (16)	0.0310 (12)	0.0239 (16)	−0.0190 (14)	0.0072 (12)
O2	0.125 (3)	0.0538 (17)	0.0457 (15)	−0.0128 (17)	−0.0233 (16)	0.0281 (14)
O3	0.153 (4)	0.094 (3)	0.108 (3)	0.067 (3)	0.014 (3)	0.049 (2)
O4	0.097 (2)	0.0467 (16)	0.0417 (14)	−0.0024 (14)	0.0025 (14)	0.0150 (13)
C1	0.067 (2)	0.0349 (18)	0.0308 (16)	0.0060 (16)	−0.0036 (16)	0.0105 (14)
C2	0.0447 (19)	0.0359 (18)	0.0297 (16)	0.0021 (14)	−0.0026 (13)	0.0090 (14)
C3	0.057 (2)	0.0401 (19)	0.0380 (18)	0.0119 (16)	0.0013 (15)	0.0168 (15)
C4	0.067 (2)	0.053 (2)	0.0384 (18)	0.0134 (18)	0.0000 (17)	0.0252 (17)
C5	0.065 (2)	0.050 (2)	0.0280 (16)	0.0102 (17)	0.0010 (15)	0.0142 (16)
C6	0.0424 (18)	0.0341 (17)	0.0315 (16)	0.0016 (13)	−0.0021 (13)	0.0148 (14)
C7	0.060 (2)	0.041 (2)	0.0445 (19)	0.0041 (16)	−0.0108 (16)	0.0180 (16)
C8	0.048 (2)	0.0393 (18)	0.0399 (18)	0.0020 (15)	−0.0030 (15)	0.0198 (15)
C9	0.080 (3)	0.040 (2)	0.051 (2)	−0.0009 (18)	−0.0083 (19)	0.0255 (18)
C10	0.101 (3)	0.052 (2)	0.046 (2)	−0.004 (2)	−0.019 (2)	0.0288 (19)
C11	0.081 (3)	0.052 (2)	0.044 (2)	−0.001 (2)	−0.0196 (19)	0.0210 (19)
C12	0.0416 (18)	0.0377 (18)	0.0389 (17)	0.0032 (14)	−0.0027 (14)	0.0200 (15)
C13	0.086 (4)	0.098 (5)	0.256 (9)	0.029 (3)	0.059 (5)	0.112 (6)
C14	0.077 (4)	0.134 (5)	0.101 (4)	−0.006 (3)	0.000 (3)	0.061 (4)
C15	0.088 (4)	0.097 (4)	0.076 (3)	0.030 (3)	0.008 (3)	0.033 (3)
C16	0.102 (4)	0.050 (3)	0.081 (3)	−0.009 (2)	0.031 (3)	0.004 (2)
C17	0.089 (4)	0.091 (4)	0.086 (3)	0.016 (3)	0.012 (3)	0.064 (3)
C18	0.080 (3)	0.055 (2)	0.0370 (19)	0.007 (2)	0.0086 (18)	0.0202 (19)

### Geometric parameters (Å, °)

**Table:**

Se1—C12	1.918 (3)	C3—C4	1.379 (5)
Se1—Se1^i^	2.3889 (8)	C3—H3	0.9300
Se2—C6	1.919 (3)	C4—C5	1.365 (5)
Se2—Se2^ii^	2.3877 (7)	C4—H4	0.9300
N1—C1	1.312 (4)	C5—H5	0.9300
N1—H1A	0.8600	C7—C8	1.484 (5)
N1—H1B	0.8600	C8—C9	1.383 (5)
N2—C6	1.336 (4)	C8—C12	1.403 (5)
N2—C5	1.346 (4)	C9—C10	1.375 (5)
N3—C7	1.330 (5)	C9—H9	0.9300
N3—H3A	0.8600	C10—C11	1.363 (5)
N3—H3B	0.8600	C10—H10	0.9300
N4—C11	1.328 (5)	C11—H11	0.9300
N4—C12	1.330 (4)	C13—H13A	0.9600
N5—C15	1.313 (6)	C13—H13B	0.9600
N5—C13	1.403 (6)	C13—H13C	0.9600
N5—C14	1.449 (7)	C14—H14A	0.9600
N6—C18	1.311 (5)	C14—H14B	0.9600
N6—C16	1.451 (5)	C14—H14C	0.9600
N6—C17	1.460 (5)	C15—H15	0.9300
O1—C1	1.233 (4)	C16—H16A	0.9600
O2—C7	1.224 (4)	C16—H16B	0.9600
O3—C15	1.210 (6)	C16—H16C	0.9600
O4—C18	1.229 (5)	C17—H17A	0.9600
C1—C2	1.487 (4)	C17—H17B	0.9600
C2—C3	1.385 (4)	C17—H17C	0.9600
C2—C6	1.406 (4)	C18—H18	0.9300
			
C12—Se1—Se1^i^	92.21 (10)	C10—C9—H9	120.1
C6—Se2—Se2^ii^	92.67 (9)	C8—C9—H9	120.1
C1—N1—H1A	120.0	C11—C10—C9	118.6 (3)
C1—N1—H1B	120.0	C11—C10—H10	120.7
H1A—N1—H1B	120.0	C9—C10—H10	120.7
C6—N2—C5	117.6 (3)	N4—C11—C10	123.3 (4)
C7—N3—H3A	120.0	N4—C11—H11	118.3
C7—N3—H3B	120.0	C10—C11—H11	118.3
H3A—N3—H3B	120.0	N4—C12—C8	122.6 (3)
C11—N4—C12	118.4 (3)	N4—C12—Se1	116.3 (2)
C15—N5—C13	123.2 (5)	C8—C12—Se1	121.1 (2)
C15—N5—C14	118.6 (5)	N5—C13—H13A	109.5
C13—N5—C14	118.2 (5)	N5—C13—H13B	109.5
C18—N6—C16	121.4 (4)	H13A—C13—H13B	109.5
C18—N6—C17	119.9 (4)	N5—C13—H13C	109.5
C16—N6—C17	118.6 (4)	H13A—C13—H13C	109.5
O1—C1—N1	121.8 (3)	H13B—C13—H13C	109.5
O1—C1—C2	119.4 (3)	N5—C14—H14A	109.5
N1—C1—C2	118.8 (3)	N5—C14—H14B	109.5
C3—C2—C6	117.2 (3)	H14A—C14—H14B	109.5
C3—C2—C1	122.8 (3)	N5—C14—H14C	109.5
C6—C2—C1	120.0 (3)	H14A—C14—H14C	109.5
C4—C3—C2	120.6 (3)	H14B—C14—H14C	109.5
C4—C3—H3	119.7	O3—C15—N5	127.4 (5)
C2—C3—H3	119.7	O3—C15—H15	116.3
C5—C4—C3	117.8 (3)	N5—C15—H15	116.3
C5—C4—H4	121.1	N6—C16—H16A	109.5
C3—C4—H4	121.1	N6—C16—H16B	109.5
N2—C5—C4	124.0 (3)	H16A—C16—H16B	109.5
N2—C5—H5	118.0	N6—C16—H16C	109.5
C4—C5—H5	118.0	H16A—C16—H16C	109.5
N2—C6—C2	122.7 (3)	H16B—C16—H16C	109.5
N2—C6—Se2	116.1 (2)	N6—C17—H17A	109.5
C2—C6—Se2	121.2 (2)	N6—C17—H17B	109.5
O2—C7—N3	122.0 (3)	H17A—C17—H17B	109.5
O2—C7—C8	120.0 (3)	N6—C17—H17C	109.5
N3—C7—C8	118.0 (3)	H17A—C17—H17C	109.5
C9—C8—C12	117.2 (3)	H17B—C17—H17C	109.5
C9—C8—C7	123.0 (3)	O4—C18—N6	125.9 (4)
C12—C8—C7	119.8 (3)	O4—C18—H18	117.0
C10—C9—C8	119.9 (3)	N6—C18—H18	117.0

Symmetry codes: (i) −*x*+1, −*y*+2, −*z*+1; (ii) −*x*, −*y*+2, −*z*+2.

### Hydrogen-bond geometry (Å, °)

**Table:**

*D*—H···*A*	*D*—H	H···*A*	*D*···*A*	*D*—H···*A*
N1—H1A···O4	0.86	2.09	2.946 (4)	170
N1—H1B···O3	0.86	2.03	2.869 (5)	163
N3—H3B···O4	0.86	2.10	2.919 (4)	158
N3—H3A···O1^iii^	0.86	2.31	3.081 (4)	150

Symmetry codes: (iii) −*x*, −*y*+1, −*z*+1.
